# Strigolactone Analogs: Two New Potential Bioactiphores for Glioblastoma

**DOI:** 10.1021/acschemneuro.1c00702

**Published:** 2022-02-09

**Authors:** Gizem Antika, Zeynep Özlem Cinar, Esma Seçen, Mehmet Özbil, Esra Tokay, Feray Köçkar, Cristina Prandi, Tugba Boyunegmez Tumer

**Affiliations:** † Graduate Program of Molecular Biology and Genetics, School of Graduate Studies, Canakkale Onsekiz Mart University, Canakkale 17020, Turkey; ‡ Graduate Program of Molecular Medicine, Universitätsklinikum Jena, Friedrich-Schiller-Universität Jena, Jena 07740, Germany; § Gebze Technical University, Institute of Biotechnology, 41400 Gebze, Kocaeli, Turkey; ∥ Department of Molecular Biology and Genetics, Faculty of Sciences and Arts, Balikesir University, Balikesir 10145, Turkey; ⊥ Department of Chemistry, University of Turin, 10125 Turin, Italy; # Department of Molecular Biology and Genetics, Faculty of Arts and Science, Canakkale Onsekiz Mart University, 17020 Canakkale, Turkey

**Keywords:** Glioblastoma, SL analogs, antiproliferative, apoptotic effect, G1-phase arrest, antiglioma effect

## Abstract

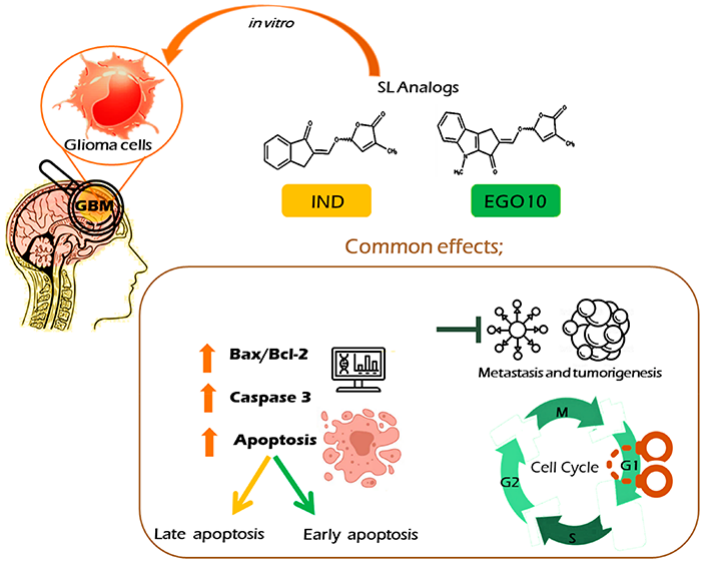

Strigolactones (SLs), carotenoid-derived phytohormones, control the plant response and signaling pathways for stressful conditions. In addition, they impact numerous cellular processes in mammalians and present new scaffolds for various biomedical applications. Recent studies demonstrated that SLs possess potent antitumor activity against several cancer cells. Herein, we sought to elucidate the inhibitory effects of SL analogs on the growth and survival of human brain tumor cell lines. Among four tested SLs, we showed for the first time that two lead bioactiphores, indanone-derived SL and EGO10, can inhibit cancer cell proliferation, induce apoptosis, and induce G1 cell cycle arrest at low concentrations. SL analogs were marked by increased expression of Bax/Caspase-3 genes and downregulation of Bcl-2. In silico studies were conducted to identify drug-likeness, blood–brain barrier penetrating properties, and molecular docking with Bcl-2 protein. Taken together, this study indicates that SLs may be promising antiglioma agents, presenting novel pharmacophores for further preclinical and clinical assessment.

## Introduction

Glioblastoma multiforme (GBM), categorized as a grade IV glioma based on the WHO classification, is the most aggressive and deadly malignant tumor originating from glial cells and their progenitor in the brain.^1^ The treatment strategies are limited due to the following reasons: (1) restriction by the blood–brain barrier (BBB) on the absorption of the chemotherapeutic agents, (2) multidrug or other intrinsic resistance mechanisms against induction of cell death, and (3) the lack of a single druggable target due to complex oncogenic pathways and genetic heterogeneity of the tumor.^2^ Therefore, new treatment strategies should focus on more potent and safer scaffolds with multitarget and BBB penetrating pharmacophores.

SL molecules include structurally and functionally diverse classes of apocarotenoids synthesized by plants and recognized/classified as novel phytohormones. Over the past few years, though it is limited, an increasing number of research studies investigating the effects of these interesting and challenging molecules on the mammalian systems have appeared in the literature (recently reviewed in ref (3)). Starting in 2012, Pollock et al. reported for the first time that some SL analogs inhibit the growth and survival of breast cancer, and shortly after they showed that these compounds are also effective on prostate, colon, lung, melanoma, osteosarcoma, and leukemic cell lines by activation of stress-related MAPKs, cell cycle arrest, and apoptosis.^4,5^ The mechanisms of action studies revealed that SLs also inhibited the growth of cells in the breast tumor xenograft model by affecting the integrity of the microtubules while normal cells were affected minimally.^6,7^ In 2018, our research group showed that a specific SL analog, GR24, promoted AKT activation in the insulin-resistant skeletal muscle cells, inhibited hepatic glucose output, and downregulated the expression of rate-limiting enzymes of gluconeogenesis-PEPCK and G6 Pase.^8^ We have also shown that GR24 is a very potent activator of the Nrf2 signaling pathway and downstream cytoprotective and phase II detoxifying enzymes.

**Figure 1:**
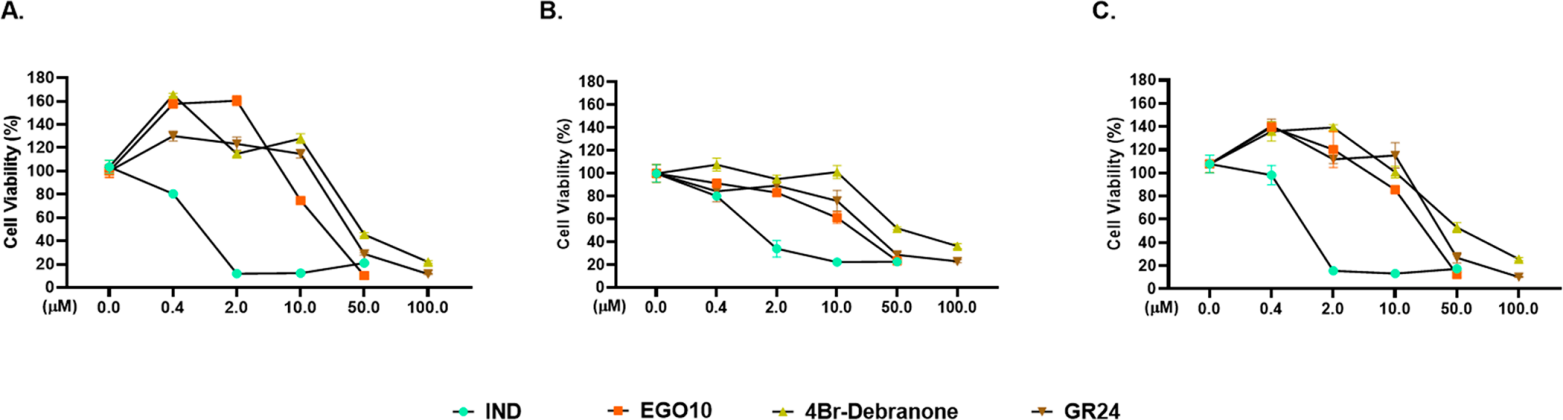
Effects of the SL analogs on the cell growth of (A) A172 and (B) U87 human glioblastoma and (C) HUVEC endothelial cell lines at the dose range of 0.4–100 μM for 72 h. Each data point is represented as the mean ± SEM obtained from three independent experiments. SEM, Standard Error of Mean. C, Control (only DMSO).

A careful assessment of published literature for the action of phytohormones (i.e., abscisic acid (ABA), cytokinins) on the mammalian central nervous system (CNS) reveals puzzling effects due to BBB penetrating ability, stress responder nature, and their roles in the redox and neurotransmitter metabolism. For example, several phytohormones (ABA, gibberellic acid, and indole acetic acid) have been detected in the brain tissue of mice after a diet enriched by these specific hormones.^9^ Interestingly, it was reported that the mammalian brain naturally contains ABA, and under stressful conditions, its serum level increases. Therefore, investigations relating the phytohormones with CNS activities are a new and interesting dimension in the literature. In 2020, our research group elucidated the effects of model SL analog GR24 on the mammalian brain under lipopolysaccharide (LPS) stress by examining microglia and BBB endothelial cells.^10^ We reported that GR24 has marked potency in the suppression of LPS induced neuroinflammatory/neurotoxic mediators by regulating NF-κB, Nrf2, and PPARγ signaling. GR24 also mitigated the LPS-increased permeability in the BBB endothelial cell line bEnd.3 through enhancing the expression of tight junction proteins.^10^ Systemic inflammation and neuroinflammation in the microenvironment of the brain have been recently accepted as unifying early onset molecular mechanisms and/or common seeds in the hardening of malignancy and metastasis processes in glioblastoma.^11^ Therefore, in light of our previous findings, we hypothesized that SLs could serve as a scaffold from which promising bioactiphores can be developed against glioblastoma. With this aim, we tested SL analogs with different pharmacophores (Table S1 in the Supporting Information). In the present work, as a first step, we evaluated the effects of four SL analogs, indanone derived SL (IND), EGO10, GR24, and 4Br-debranone (see Table S1 for the IUPAC names), on the growth of human glioma cell lines U87 and A172 and human normal endothelial cell line HUVEC (as a control) by SRB cytotoxicity assay. All cells were treated with each of these SL analogs at a dose range of 0.4–100 μM or DMSO (0.1%) as a vehicle for 24, 48, and 72 h incubation periods. According to dose–effect curves, although all SL analogs significantly inhibited the brain cancer cell growth at different concentrations over each time (Figure 1 for 72 h, Figure S2 for 24, and Figure S3 for 48 h), the most potent results as antiproliferative agents were detected for IND and EGO10 on both U87 and A172 cells. The half-maximal IC_50_ of IND, after 24, 48, and 72 h in A172, is 2.5, 2.8, and 0.8 μM; in U87 it is 17, 1.1, and 1.2 μM; and in HUVEC it is 4.4, 2.1, and 2.9 μM, respectively (Table S2 in the Supporting Information). Natural SLs have a general structure with a core tricyclic ABC lactone ring system attached to a butenolide D-ring by an enol-ether bridge (see Table S1 for representation).^12^ IND is a synthetic SL analog derived from indanone with B, C, and D ring systems,^12^ and it has never been examined on a mammalian cell system including cancer models (see the spectral properties of IND in Figure S1 in the Supporting Information). All of the tested molecules have been used as racemic mixtures.

EGO10 suppressed the cell proliferation of gliomas for 48 h (IC_50_ for A172 and U87 were 15.3 and 14.0 μM, respectively) and 72 h (IC_50_ for A172 and U87 were 17.1 and 17.5 μM, respectively) in a very similar dose-dependent manner (Figure 1 and Table S2 (in the Supporting Information)). In the study of Pollock et al., EGO10, which was abbreviated as EGO9C, was reported to suppress the growth and survival of an array of cancer cell lines including prostate (PC3 and DU145), colon (HT-29, HCT116, and SW480), and lung (A549) cancer with IC_50_ values higher than 48 μM.^5^ Thus, glioma cells could be more sensitive to EGO10, and together with IND, they might be promising candidates against human glioblastoma. Although the dose–response curves and IC_50_ values of GR24 and 4Br-debranone represented variable inhibitory effects for the growth of U87 and A172 cells, IND and EGO10 had quite lower IC_50_ values and similar sensitivity on both glioblastoma cell lines. Therefore, we chose these two SL analogs as the lead compounds to offer further insight into their mechanism of action.

**Figure 2:**
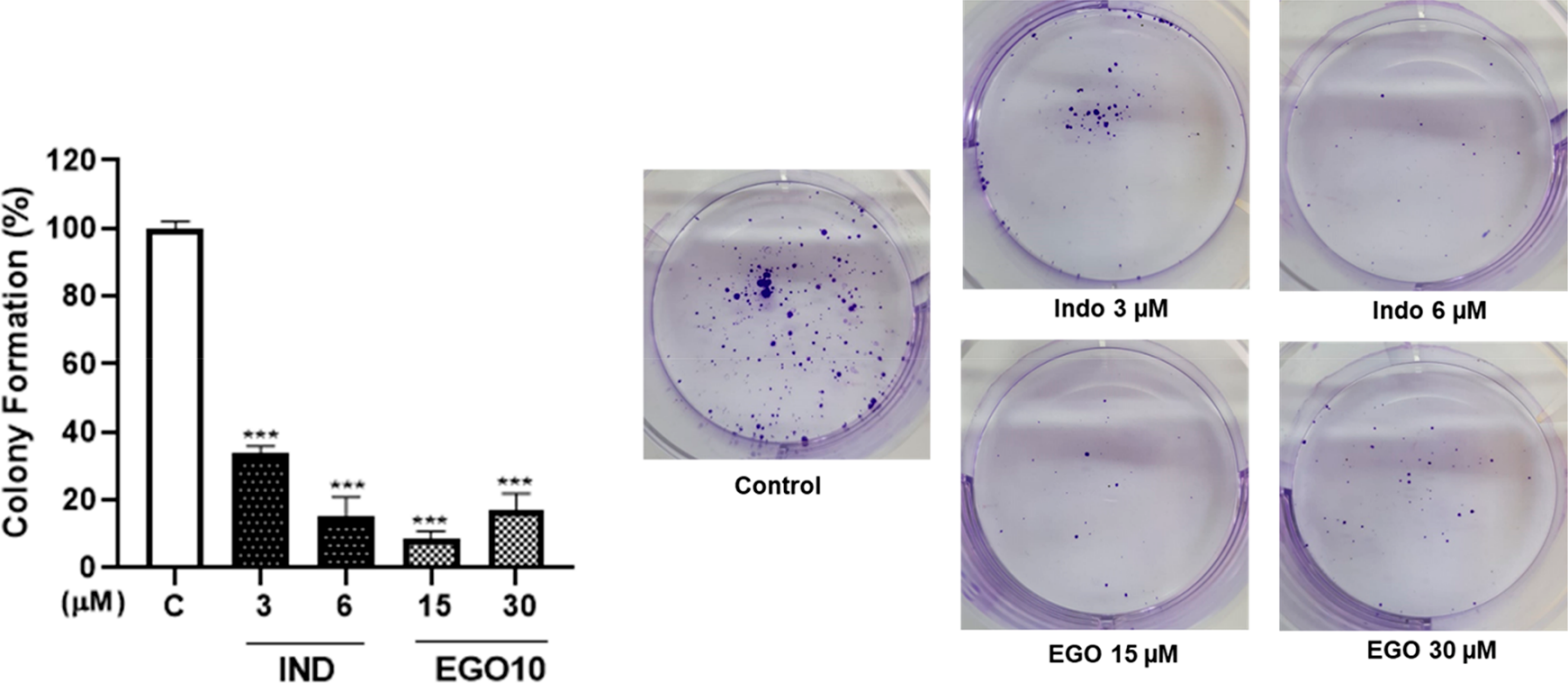
Variation in the colony formation capacity of A172 cells after IND (3–6 μM) and EGO10 (15–30 μM) treatments. Data are presented as mean ± SEM of three independent experiments conducted in duplicate. C: Control (only DMSO). ***p < 0.001.

We evaluated the antitumorigenic potential of IND and EGO10 at very low doses in the A172 cell line by colony formation assay, which also confirmed the long-lasting effects of the compounds on reducing cell viability. IND at doses of 3 and 6 μM strongly and dose-dependently inhibited the percentage of colony formation capacity in A172 cells by 66% and 85%. In the case of EGO10, at 15 μM, the proliferation ability of A172 cells was almost completely (91%) suppressed (Figure 2).

**Figure 3:**
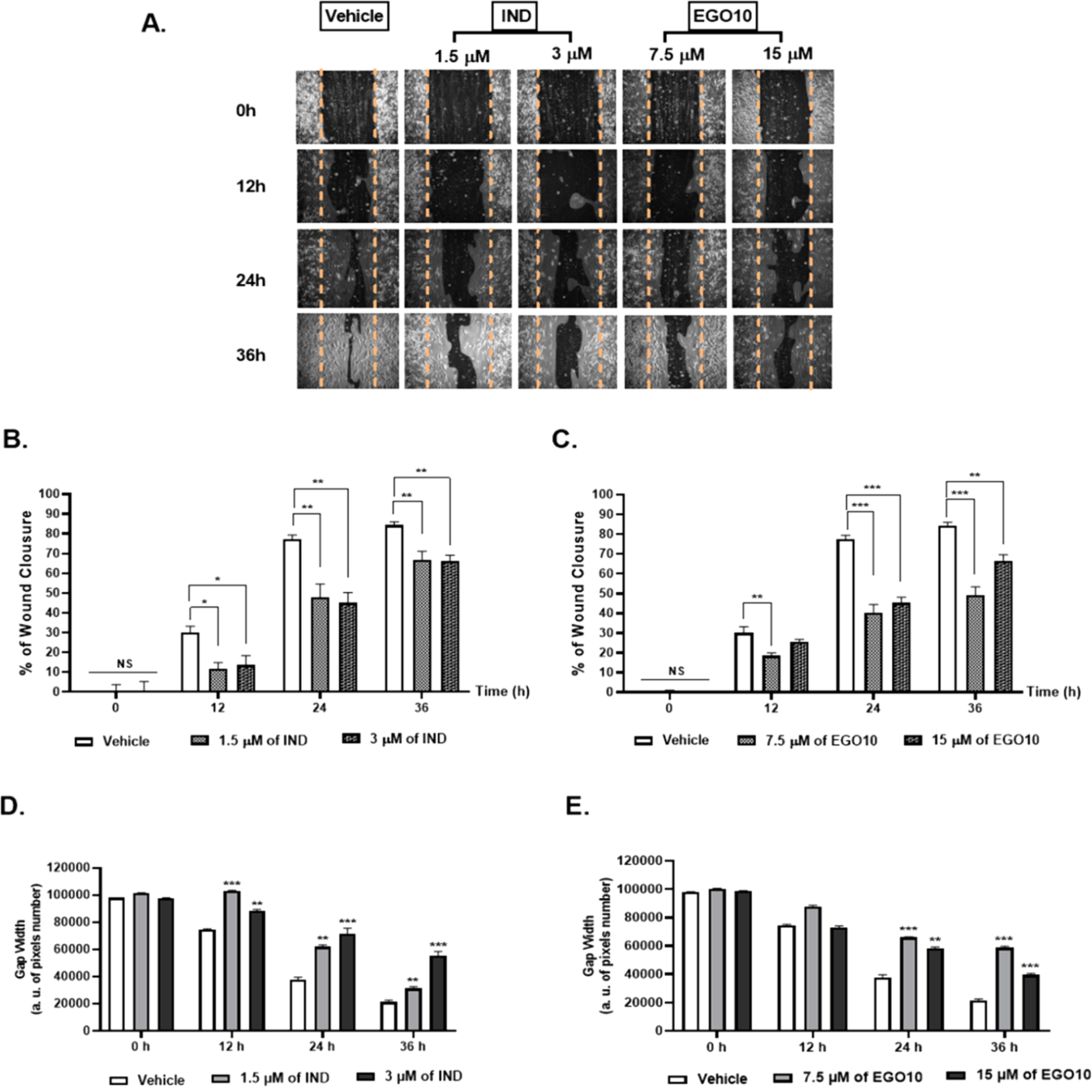
EGO10 and IND suppressed the cell migration ability of A172 glioblastoma cells. (A) Representative images of A172 cells and the wound healing measurements including wound closure area and gap width for the treatment of IND and EGO10 at different concentrations. V: Vehicle (only DMSO). **p < 0.005, ***p < 0.001.

To evaluate the effects of IND and EGO10 on A172 migration capacity, a wound-healing assay was carried out. Accordingly, 1.5 and 3 μM doses of IND significantly reduced the wound closure rate in A172 cells by 61% and 55%, respectively, as compared to the vehicle group at 12 h (Figure 3B). After 24 h, IND significantly inhibited the migration of A172 cells at the same doses by 38% and 42%, respectively, in comparison to the vehicle group (Figure 3A and B). Although the wound area of control (vehicle) was already closed at the end of 36 h, IND-treated cells at both doses showed a similar and significant difference (21%) in the suppression of wound closure. Moreover, the cumulative narrowing gap obtained in the control was significantly and time-dependently prevented by 1.5 and 3 μM of IND at all time points, which was a 1.4- and 1.3-fold longer gap at 12 h, 1.7- and 2.0-fold at 24 h, and 2.1- and 2.2-fold at 36 h, respectively (Figure 3A and D). EGO10 at 7.5 μM significantly and time-dependently suppressed the closure rate of the wound in A172 cells as 38% at 12 h, 48% at 24 h, and 42% at 36 h (Figure 3A and C). The gap width of the wound scratched on A172 cells, which were treated with 7.5 μM of EGO10, showed a significantly forceful inhibitory effect on the migration of glioma cells by a 1.8- and 2.6-fold longer gap than the control at 24 and 36 h, respectively (Figure 3E). These results suggest that IND and EGO10 may have the ability to inhibit the invasiveness of glioblastoma cells at very low concentrations.

The study of Mayzlish-Gati et al. showed that two SL analogs, MEB55 and ST362, affected the integrity of the microtubular network in the MDA-MB-231 breast cancer cell line.^6^ In this context, the mechanism underlying the inhibitory effects of IND and EGO10 on glioblastoma cell migration might be related to their effects on the integrity of the microtubular network. However, this should be verified with further analyses for both SL analogs due to their quite different structures from those mentioned above.

**Figure 4:**
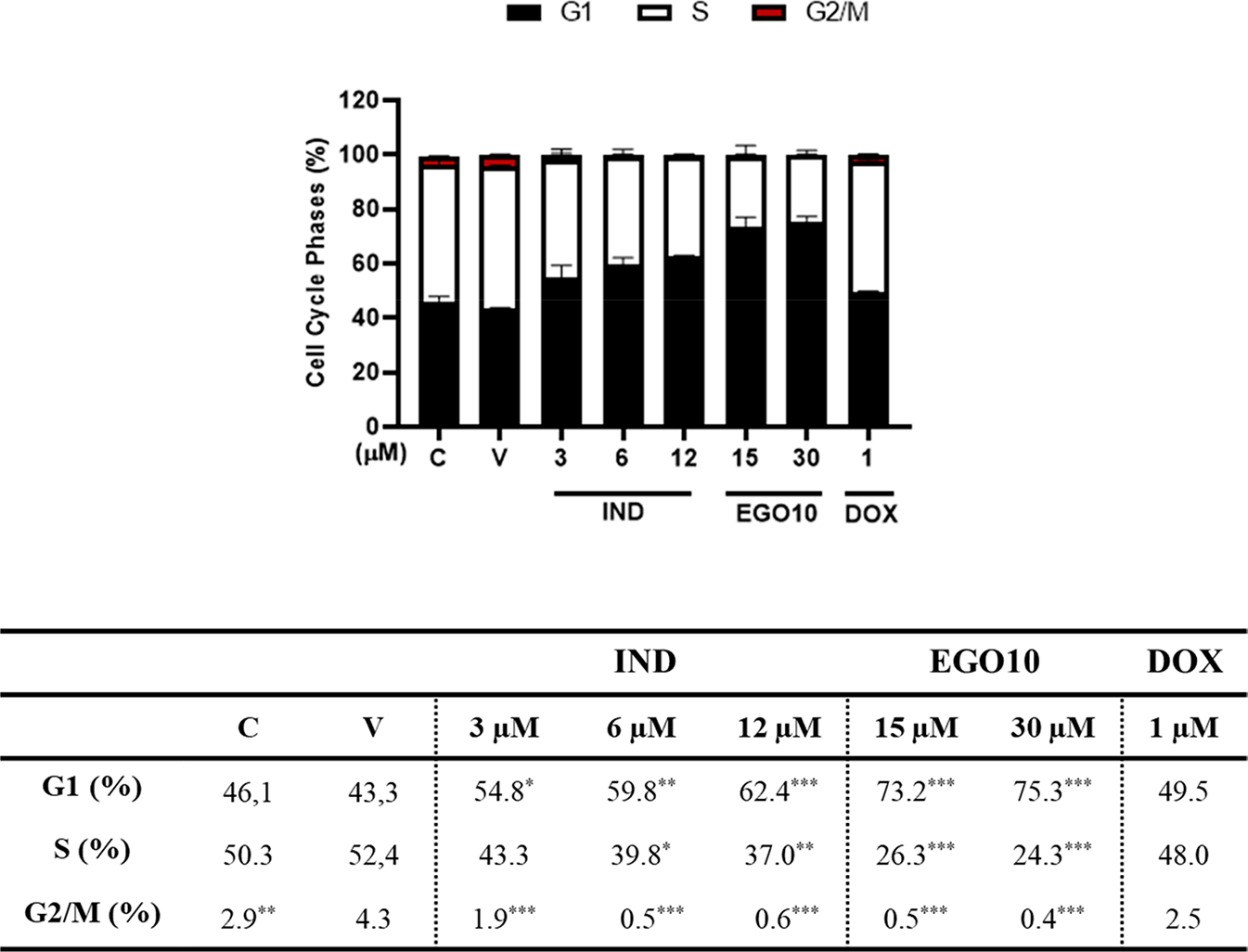
EGO10 and IND induce the G1 arrest of the cell cycle on A172 cells at 48 h according to the cell cycle arrest assay. C: Control (nontreated). V: Vehicle (only DMSO). DOX: positive control as a chemotherapeutic drug. *p < 0.02, **p < 0.005, ***p < 0.001.

To further investigate the mechanism(s) of action responsible for the potent reduction in cellular growth and survival by very low doses of IND and EGO10 in A172 glioma cells, we examined whether such a reduction was associated with a cytotoxic effect due to changes in cell cycle progression. Pollack et al. showed that GR24 inhibited the mammosphere formation and growth of breast cancer by inducing apoptosis through the G2/M phase arrest for 72 h.^4^ In addition, the G2/M phase of cell cycle arrest was significantly promoted on colon and prostate cancer cells by the treatment of SL analogs: ST357, ST362, and MEB55 for 48 h.^5^ In our study, the two SL analogs with different pharmacophores lead to G1-arrest in A172 glioma cells. The cell cycle analysis of A172 cells treated with 15 and 30 μM of EGO10 indicated a significant increase in the G1 phase of the glioblastoma cells (from 47% in the vehicle to 73.2% and 75.3%, respectively) at 48 h (Figure 4). Moreover, the G1 arrest of the cell cycle was also induced by 6 μM of IND as well as more significantly at a 12 μM concentration as compared to the control. It is known that mutations in p16 or p19 genes lead to abnormalities in cell cycle progressions such as disruption of G0/G1 arrest and cyclin-dependent kinase expression and in the production of epidermal growth factor receptors in GBM.^13^ The diverse effects of the SL analogs on cell cycle arrest might be related with the cancer cell-specific mutations and promoting factors. Therefore, these results point to cytotoxic effects of IND and EGO10 on A172 glioma cells and suggest their anticancer effects as putative adjuvant treatment. Although varying concentrations of both EGO10 and IND resulted in remarkable changes in the cell cycle distribution, there was no significant change in the percentage of the cell cycle phases in the cells treated with 1 μM of doxorubicin (DOX, a chemotherapeutic drug) relative to the control (Figure 4). Similar to our results obtained for DOX treatment in A172 cells, in D54 and GBM6 glioblastoma cell lines treatment with DOX at 0.125 μM leads to comparable cell cycle distribution data with the untreated control cells.^14^

**Figure 5:**
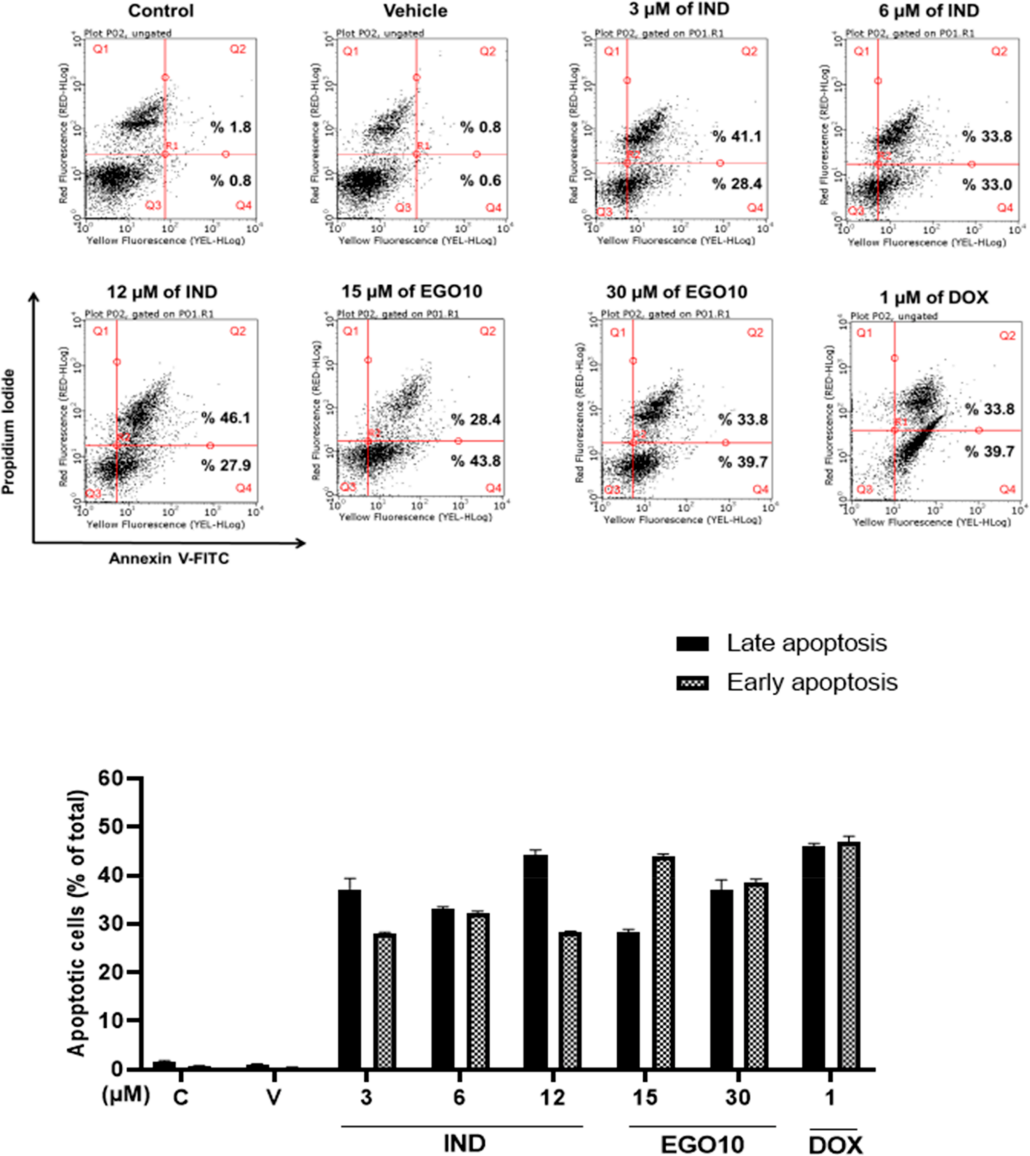
Annexin V/PI staining of A172 cells after treatment with IND and EGO10 for 48 h. The apoptotic cell ratios were demonstrated as the early (Annexin-/PI+) and late (Annexin+/PI+) apoptosis percentage bars. Q1, Q2, Q3, and Q4 quadrants are represented as the areas of necrosis, late apoptosis, viable cells, and early apoptotic cells, respectively. C: Control (nontreated). V: Vehicle (only DMSO).

For the mechanism of action studies, we next investigated whether EGO10 and IND induced apoptosis in A172 glioma cells. With this aim, A172 cells were treated with IND (3, 6, and 12 μM) and EGO10 (15 and 30 μM) for 48 h and then stained by annexin V–FITC (annexin V) to analyze the apoptotic cell ratios by flow cytometry. As illustrated in Figure 5, both IND and EGO10 at their IC_50_ concentrations effectively increased the apoptotic cell percentage as compared to control. Previously, in the study of Pollock et al., 15 and 20 μM of EGO10 (coded as EGO9C) induced the late apoptotic events in HCT116 colon cells.^5^ However, in the current study, EGO10 stimulated the apoptotic cell death in glioma cells more prominently at the early apoptotic stage (Q4) by 43.8% at 15 μM and 39.7% at 30 μM, which is the same effect detected after the treatment with 1 μM DOX relative to the vehicle (0.6% at Q4; Figure 5). In the case of IND, 3 and 12 μM treatment doses resulted in remarkable increases for the late apoptotic cell death by 41.1% and 46.1%, respectively, as compared to that of the vehicle (0.8% in Q2, late apoptosis quaternary).

**Figure 6:**
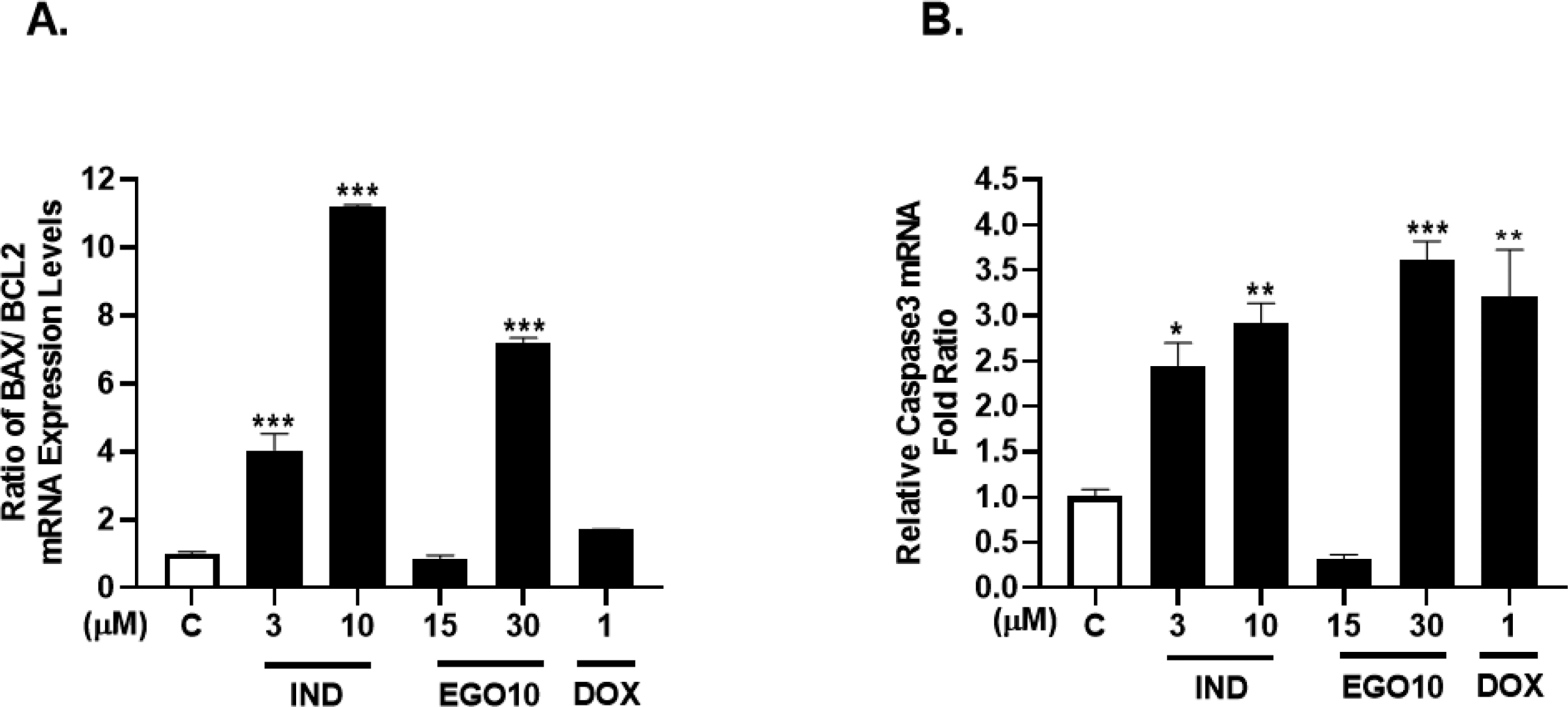
SL analogs increased the (A) Bax/Bcl-2 ratio and (B) Caspase-3 in mRNA expression level in A172 glioblastoma cells. C: Control (only DMSO). DOX: positive control. *p < 0.02, **p < 0.005, ***p < 0.001.

In the molecular mechanism of the apoptotic cell death program, a shift in Bax/Bcl-2 expression ratio at the transcriptional level initiates apoptosis by activating Caspase-3, as was reported in various tumors.^15^ In the early stage of the apoptosis mechanism, activation of the p53 tumor suppressor leads to the expression of pro-apoptotic mediators including the Bax/caspase-3 and the inhibition of Bcl-xL/Bcl-2. Activation of caspase-3 and caspase-7 stimulates endonucleases to break DNA in the late phase of apoptosis.^16^ In A172 cells, treatments with IND at 3 and 10 μM strongly and significantly increased the Bax/Bcl-2 ratio at the mRNA expression level by 4- and 11.2-fold, respectively, while 30 μM of EGO10 enhanced the ratio by 7.2-fold compared to the control (Figure 6A). Additionally, DOX at 1 μM increased the ratio by 1.7-fold as compared to the control, which was found as a mild modulatory effect on Bax and Bcl-2 gene expressions compared to these two SL analogs in A172 cells. Moreover, 3 and 10 μM of IND significantly increased the gene expression level of caspase-3 by 2.4- and 2.9-fold, respectively, while EGO10 at 30 μM enhanced the level by 3.6-fold relative to the control (Figure 6B). As a result, IND and EGO10 at higher doses represented a remarkable increase in Bax/Bcl-2 and caspase-3 gene expressions while inducing the upregulation in the late apoptosis ratio.

The pharmacokinetic properties were calculated for both EGO10 and IND compounds. These calculated properties are provided in Table S3 in the Supporting Information. Both molecules were water-soluble (IND with slightly higher solubility), and they were both druggable according to Lipinski and Veber druggability (both showed 0 violations for Lipinski rule of 5), which are the most widely used filters for evaluating drug-likeness. Both molecules were BBB permeable and could be absorbed by the gastrointestinal system. These molecules were not p-glycoprotein substrates or inhibitors. We also computationally investigated the binding of IND and EGO10 to the Bcl-2 protein for testing their ability to bind and possibly inhibit Bcl-2. Molecular docking simulations revealed that both molecules yielded higher binding affinities than the previously reported inhibitor molecule, 4FC, which was cocrystallized via a solution NMR technique.^17^ Binding affinities were provided in Table S4 in the Supporting Information.

Both ligands were bound to the same binding pocket as 4FC in the solution NMR structure (Figure S4 in Supporting Information). EGO10 yielded 1.83 kcal/mol higher binding affinity than 4FC, whereas IND yielded 0.85 kcal/mol higher binding affinity. Although the 0.85 kcal/mol difference is very small in molecular docking simulations, IND still binds slightly stronger than 4FC. The binding pockets for both IND and EGO10 were very similar to that of the 4FC molecule present in the solution NMR structure (PDB ID: 1ysg). Interacting residues for both molecules were provided in Figure S5 in the Supporting Information. The higher binding affinity provided by EGO10 was a result of increased π–π interactions with the protein because it contained the tricyclic lactone group. EGO10 interacted with Bcl-2 protein through π–π stacked and π–π T-shaped interactions through amino acids Tyr 189 and Phe 101, whereas IND only interacted with Tyr 199 through π–π stacked interactions. Moreover, EGO10 interacted with Ala 97 residue through two π-alkyl interactions but IND only with one π-alkyl interaction. 3-D representations are provided in Figure S4 in the Supporting Information.

Following molecular docking calculations, the best binding poses for EGO10 and IND were subjected to 30-ns-long molecular dynamics (MD) simulations. Both EGO10 and IND stayed in very similar binding pockets obtained from molecular docking simulations. In the first 10 ns period of the simulation, EGO10 moved away from the binding site with a calculated root-mean-square deviation (RMSD) value of 12.72 Å. Then, EGO10 was stabilized in the initial binding pocket and stayed there for the rest of the simulation. The RMSD value for EGO10 from the second 10 ns period was 5.97 Å, and it was 1.39 Å from the last 10 ns period. Likewise, IND stayed at the binding pocket obtained from the molecular docking simulations throughout the MD simulations. The RMSD value for IND was calculated as 3.30 and 2.32 Å for the whole 30 ns time frame and the last 10 ns period of the simulations, respectively. Time evolutions of EGO10-Bcl2 and IND-Bcl-2 complexes are provided in Figure S6 and Figure S7, respectively, in the Supporting Information.

Binding free energies for both ligands were calculated, and IND produced 27.16 kcal/mol lower binding energy than EGO10, which was contradicting the slightly higher binding affinity (0.98 kcal/mol) for EGO10. When the interaction map provided in Figure S8 in the Supporting Information was analyzed, it was clear that EGO10 lost its π–π stacked and π–π T-shaped interactions through Phe 101 and Tyr 199 of Bcl-2, whereas IND kept most of its interactions from the docking pose. Moreover, IND increased the number of hydrophobic interactions compared to docking interactions. Thus, IND interacted with Bcl-2 with much higher binding free energy.

Herein, we have elucidated that the mechanisms of both IND and EGO10 on the inhibition of glioma cancer cell growth involve both interferences with cell cycle progression and apoptosis. Overall, in this study, findings suggest that in the treatment and prevention of glioma, EGO10 and indanone-derived SL may provide a novel multipotent therapeutically active structure, on which in silico optimized SL-like pharmacophores can be developed and synthesized for further preclinical and clinical investigations.

## Methods

### Chemicals

(±) GR24, (±) EGO10, 4Br-debranone were purchased from Strigolab. Indanone-derived SL (IND) was synthesized according to the procedure reported in ref (12).

### Cell Culture

A172 (human glioblastoma cells), U87 (human primary glioblastoma cells), and HUVEC (human umbilical vein endothelial cells) cell lines were cultured and maintained in high glucose DMEM supplemented with 100 U/mL penicillin-streptomycin and 10% FBS. Cells were incubated at 37 °C in humidified air containing 5% CO_2_.

### Cell Viability Assay

A172 and U87 glioblastoma and HUVEC endothelial cells were seeded into 96-well plates at concentrations of 5 × 10^4^ cells per well and incubated for 24 h. The SL analogs were solubilized in dimethyl sulfoxide (DMSO, sc-358801, Santa Cruz). Cells were treated with various doses (between 1 to 100 μM) of SL analogs (IND, EGO10, 4-debranone, GR24) and incubated for 24, 48, and 72 h separately. The effects of the compounds on cell viability were determined by sulforhodamine B (SRB) assay as described previously.^18^

### Wound Healing Assay

The wound-healing assay was performed to illustrate the change in the migration capacity of glioblastoma cells by the treatment of SL analogs. A172 cells were treated with IND (1.5 μM and 3 μM) and EGO10 (7.5 μM and 15 μM). After 48 h of incubation with SL analogs, the cells were scrapped in the middle of a well from up to the bottom side by using a 200 μL pipette tip to create a wound. Every sample was photographed at 10× magnification five times using Launch ImageFocus 4 software associated with the inverted microscope (Euromex) at 0, 12, 24, and 36 h. The gap width and wound closure areas of each image were measured using ImageJ software.^19^

### Colony Formation Assay

The effects of IND and EGO10 on A172 and U87 cell proliferation were also analyzed by colony formation assay. A172 and U87 cells were treated with specified doses of SL analogs. After 48 h of incubation, cells were trypsinized to seed into a new six-well plate at a concentration of 1 × 10^3^ cells per well in duplicate for each treatment group. Growth media of cells were refreshed periodically during 20 days of incubation. Cells were fixed with 10% formaldehyde for 30 min, and then fixed colonies were stained via 1% crystal violet for 5 min at RT. Then, each well was washed with PBS three times. The colonies were counted by using a cell counter plug-in by ImageJ software.

### Gene Expression Analysis by Quantitative PCR

The total RNA samples were isolated by using a NORGEN Total RNA Purification Plus Kit, and their concentrations were measured with a QubitTM RNA BR Assay Kit according to the protocols presented by the manufacturer. cDNA synthesis of these RNA samples was performed by using the High Capacity cDNA Reverse Transcription Kit. The quantitative gene expression levels of Bax, Bcl-2, and Caspase-3 were analyzed using specific TaqMan Gene Expression Assay probes and TaqMan Fast Advanced Master Mix by using Applied Biosystems 7500 Real-Time PCR Systems (Applied Biosystems, Thermo Fisher Scientific) as described in our previous study.^8^ All gene expression levels were normalized to β-actin expression levels as a housekeeping gene. The effects of different doses of compounds on gene expression levels were evaluated via the comparative ΔΔCt method.

### Flow Cytometry Analysis for Cell Cycle Arrest and Apoptosis

A172 cells were treated with EGO10 and IND for 48 h. The cells were collected with Trypsin–EDTA. The pellet was washed with PBS, and then the cells were stained with FITC-conjugated Annexin-V and propidium iodide (PI), using an Annexin-V-FITC apoptosis detection kit according to the manufacturer’s recommendation (Calbiochem). Samples were analyzed using a flow cytometer and analyzed with CPX software (Beckman Coulter FC500 System, USA). Each assay was repeated at least two times.

### In Silico Analysis

#### Molecular Docking Simulations

Pharmacokinetic properties were predicted using the SwissADME Web server (SwissADME: a free web tool to evaluate pharmacokinetics, drug-likeness, and medicinal chemistry friendliness of small molecules).^20^

Molecular docking simulations were performed using YASARA Structure software.^21^ This software utilizes Autodock Vina for performing molecular docking simulations.^22^ The ligand bound Bcl-2 structure was used as the initial 3-D structure (PDB ID: 1ysg).^17^ This structure contained two ligands, 4FC and TN1. For validation of our simulations, first these two molecules were docked to Bcl-2 protein. We obtained very similar binding modes compared to the NMR solution structure with RMSD values of 1.07 and 0.49 Å for molecules 4FC and TN1, respectively. Their calculated binding affinities were −7.73 kcal/mol for 4FC and −6.33 kcal/mol for TN1 molecule. After validation, we docked IND and EGO10 to the Bcl-2 protein. We obtained a very similar binding mode to the 4FC molecule (Figure S3 in the Supporting Information). This site is a very common binding pocket for small molecule inhibitor candidates. All ligands were subjected to energy minimization in YASARA Structure software utilizing the Amber 03 force field.^23^ Postdocking analysis and visualization were performed with built-in tools in YASARA Structure. A 2-D interaction scheme was prepared using Discovery Studio 2021 Client software (BIOVIA, Dassault Systèmes, Discovery Studio Client, San Diego: Dassault Systèmes, 2021).

#### Classical Molecular Dynamics (MD) Simulations

Best docking poses for EGO10 and IND, with the highest binding affinity, were subjected to classical molecular dynamics (MD) simulations to assess the stability of the ligands within these binding pockets. YASARA Structure software was utilized with the AMBER03 force field,^23,24^ and the simulation length was 30 ns for each simulation replica. Simulations were replicated twice, and as the structural and statistical data were very similar to each other, data only from one replica were discussed throughout the manuscript. EGO10 and IND bound Bcl-2 proteins were placed in truncated, cubic boxes with dimensions of 76.46 Å × 76.46 Å × 76.46 and 73.88 Å × 73.88 Å × 73.88 Å, respectively. These dimensions ensured that, at any point of the simulation, the proteins stayed in the simulation box. Single point charge (SPC) water molecules^25^ were placed into the box, and sodium and chloride ions were added to neutralize the system. First, the starting system was subsequently energy-minimized using the steepest descent method for 50 000 steps. Then, energy-minimized structures were taken for the production phase. MD simulation without any constraints was carried out using a constant number of particles (*N*), pressure (*P*), and temperature (*T*), i.e., the NPT ensemble. The SETTLE algorithm was utilized to constrain the bond length and bond angle of the water molecules,^26^ while the LINCS algorithm was used to constrain the bond length of the peptide.^27^ The particle-mesh Ewald (PME) method was utilized to treat long-range electrostatic interactions.^28^ A constant pressure of 1 bar was applied with a coupling constant of 1.0 ps, and water molecules/ions were coupled separately to a bath at 298.15 K with a coupling constant of 0.1 ps. The equation of motion was integrated at 2 fs time steps using a leapfrog algorithm.^29^ The tools available in YASARA Structure software were utilized to analyze trajectories.

Binding free energies for each ligand were also calculated utilizing the md_analyzebindingenergy macro.^30^ This macro module calculates binding free energies without the entropy term.
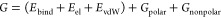


Here, the first three terms represent binding, electrostatic, and van der Waals interactions, respectively. *G*_polar_ and *G*_nonpolar_ represent polar contributions and nonpolar contributions of solvation free energies, respectively. It is very similar to the MM/PBSA for the entropy term, which can be neglected in the context of the main goal of these calculations. Then, binding free energy is calculated by the following equation:
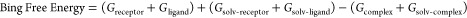


*G*_receptor_ defines the potential energy of the receptor, and *G*_ligand_ defines the potential energy of the ligand. The next two terms define solvation energies of receptor and ligand; the last two terms are potential and solvation energies of the complex.

#### Statistical Analysis

Data were analyzed by utilizing GraphPad Prism 8 software and are expressed as mean ± SEM of three independent biological replicates, and differences were analyzed by one-way ANOVA with Dunnett’s Post Hoc Test (*p < 0.02, **p < 0.005, ***p < 0.001).

## Supporting Information Available

The chemical structures and IUPAC names of SL analogs, the effects of these SLs on A172, U87, and HUVEC cell lines for 24 and 48 h and *in silico* analysis (PDF)
