# Supplementary material for: Strigolactone Analogs: Two New Potential Bioactiphores for Glioblastoma
Source: ACS Chem Neurosci. 2022 Feb 9;13(5):572–80. doi: 10.1021/acschemneuro.1c00702 (PMC8895406; doi:10.1021/acschemneuro.1c00702)
Supplement: Supplementary file 1 — cn1c00702_si_001.pdf [file cn1c00702_si_001.pdf]

Supporting Information

Strigolactone Analogs: Two New Potential Bioactiphores for Glioblastoma

Gizem Antika[1], Zeynep Özlem Cinar[1], Esma Seçen[2], Mehmet Özbil[3], Esra Tokay[4], Feray Köçkar[4], Cristina Prandi[5], Tugba Boyunegmez Tumer*[6]

[1] Graduate Program of Molecular Biology and Genetics, School of Graduate Studies, Canakkale Onsekiz Mart University, Canakkale 17020, Turkey

[2]Graduate Program of Molecular Medicine, Universitätsklinikum Jena, Friedrich-Schiller-Universität Jena, Jena 07740, Germany

[3] Gebze Technical University, Institute of Biotechnology, 41400 Gebze, Kocaeli, Turkey

[4] Department of Molecular Biology and Genetics, Faculty of Sciences and Arts, Balikesir University, Balikesir 10145, Turkey

[5] Department of Chemistry, University of Turin, 10125 Turin, Italy

[6] Department of Molecular Biology and Genetics, Faculty of Arts and Science, Canakkale Onsekiz Mart University, 17020 Canakkale, Turkey

*Corresponding Author:

Tugba Boyunegmez Tumer, E-mail: tumertb@comu.edu.tr, tumertb@gmail.com

List of Contents

Table S1. The chemical structures of four SL analogs used in this study……………………………………...S3

Table S2. IC_{50} concentrations of four SL analogs on different glioblastoma and HUVEC cell lines over time...S3

Table S3. Pharmacokinetic properties and druggability of EGO10 and IND molecules……………..................S4

Table S4. Binding affinities of IND, EGO10, and 4FC were calculated from molecular docking calculations and binding free energies calculated from molecular dynamics simulations.…………...S4

Figure S1. Spectroscopic properties of IND including *A.* H1 and *B.* C13 NMR analysis.............................S5-S6

Figure S2. Effects of the SL analogs on the cell growth of *A.* A172 and *B.* U87 human glioblastoma and *C.* HUVEC endothelial cell lines at the dose range of 0.4-100 µM for 24 h…...………………………...….……S6

Figure S3. Effects of the SL analogs on the cell growth of *A.* A172 and *B.* U87 human glioblastoma and *C.* HUVEC endothelial cell lines at the dose range of 0.4-100 μM for 48 h……….…………..………...….................…..S6

Figure S4. A) Ligands (indanone-derived SL (IND) in red, EGO10 in orange) positioned in the same binding pocket after molecular docking simulations were visualized, superimposed with the crystal structure ligand, 4FC (magenta).………………..…………………………………………………………………………………...S7

Figure S5. 2-D interaction schemes for IND and EGO10 with Bcl-2 protein obtained from molecular docking simulations…………………………………………………………………………………………………..S7

Figure S6. Time evolution of EGO10-Bcl2 complexes throughout MD simulations. EGO10 stayed at the very similar binding site obtained from molecular docking simulations throughout MD simulations.……………………………………………………………………………………………………S8

Figure S7. Time evolution of IND-Bcl2 complexes throughout MD simulations. IND stayed at the very similar binding site obtained from molecular docking simulations throughout MD simulations.……………………………………………………………………………………………………S9

Figure S8. 2-D interaction schemes for EGO10 and IND with Bcl-2 protein obtained from molecular dynamics simulations.……………………………………………………………………………………………………S9

Table S1. The chemical structures of four SL analogs used in this study.

Chemical Structure	Compound	IUPAC Name
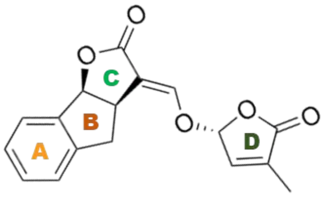	GR24	(±) (3a*R*,8b*S*,*E*)-3-((((*R*)-4-methyl-5-oxo-2,5-dihydrofuran-2-yl)oxy)methylene)-3,3a,4,8b-tetrahydro-2*H*-indeno[1,2-*b*]furan-2-one
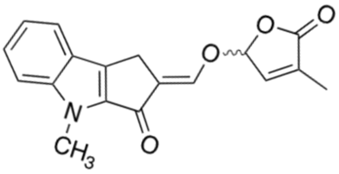	EGO10	(±)(*E*)-4-methyl-2-(((4-methyl-5-oxo-2,5-dihydrofuran-2-yl)oxy)methylene)-1,4-dihydrocyclopenta[*b*]indol-3(2*H*)-one
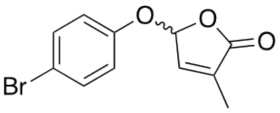	4Br-debranone	(±) 5-(4-bromophenoxy)-3-methylfuran-2(5*H*)-one
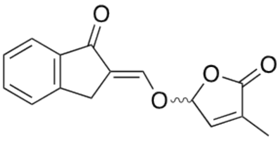	IND	(±) (*E*)-3-methyl-5-((1-oxo-1,3-dihydro-2*H*-inden-2-ylidene)methoxy)furan-2(5*H*)-one

Table S2. IC_{50} concentrations of four SL analogs on different glioblastoma and HUVEC cell lines over time. The values are expressed as the mean ± SE of three independent experiments.

	IC_{50} (µM) Values of SL analogs on Cell Growth								
**Time (h)**	**24 h**			**48 h**			**72 h**		
**Cell Lines**	**A172**	**U87**	**HUVEC**	**A172**	**U87**	**HUVEC**	**A172**	**U87**	**HUVEC**
SL ANALOGS	**IC_{50} ± SE (µM)**			**IC_{50} ± SE (µM)**			**IC_{50} ± SE (µM)**		
**IND**	2.5 ± 1.0	17.0 ± 0.9	4.4 ± 1.3	2.8 ± 0.2	1.1 ± 0.1	2.1 ± 1.3	0.8 ± 0.1	1.2 ± 0.2	2.9 ± 1.5
**EGO10**	28.5 ± 4.3	64.8 ± 17.0	37.7 ± 1.2	15.3 ± 1.8	14.0 ± 3.5	20.1 ± 1.2	17.1 ± 0.2	17.5 ± 3.2	21.6 ± 1.1
**4Br-debranone**	65.6 ± 7.3	154.4 ± 23.2	92.7 ± 1.1	44.7 ± 11.7	54.0 ± 7.1	73.7 ± 1.1	47.1 ± 9.0	67.8 ± 5.2	55.7 ± 1.1
**GR24**	96.9 ± 6.8	69.5 ± 11.3	62.7 ± 1.1	59.9 ± 3.4	26.2 ± 2.3	40.6 ± 5.0	38.0 ± 9.0	25.1 ± 2.9	31.1 ± 1.1

Table S3. Pharmacokinetic properties and druggability of EGO10 and IND molecules.

	Water solubility	Lipinski druggability	Veber druggability	BBB permeability	Human intestinal absorption	p-glycoprotein substrate	p-glycoprotein inhibitor
**IND**	Soluble (-3.17 logS)	Yes	Yes	Yes	High	No	No
**EGO10**	Soluble (-3.66 log S)	Yes	Yes	Yes	High	No	No

Table S4. Binding affinities of IND, EGO10, and 4FC were calculated from molecular docking calculations and binding free energies calculated from molecular dynamics simulations.

	IND	EGO10	4FC
**Binding Affinities (kcal/mol)**	-8.58	-9.56	-7.73
**Binding Free Energies (kcal/mol)**	-60.37	-33.21	NA

A.

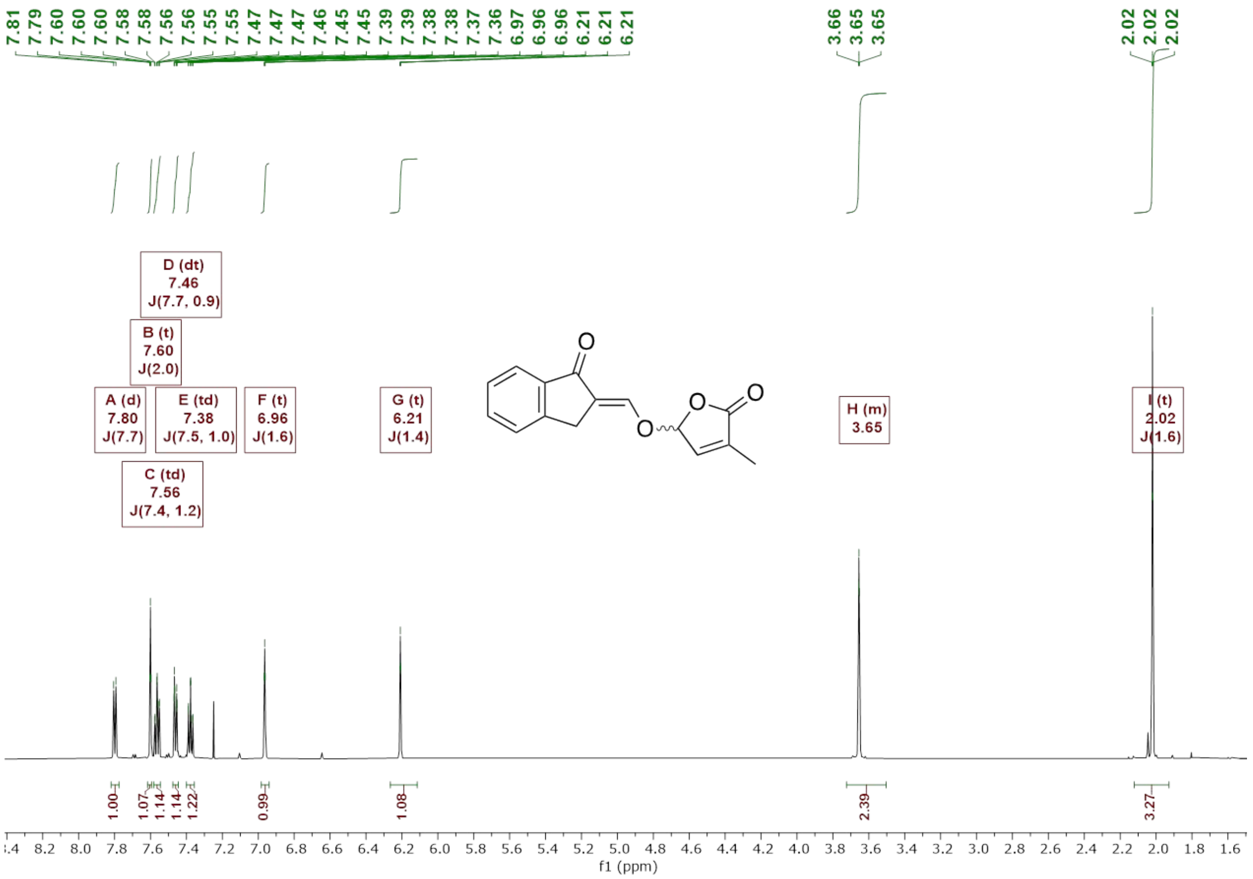
D (dt)
7.46
J(7.7, 0.9)
B (t)
7.60
J(2.0)
A (d)
7.80
J(7.7)
E (td)
7.38
J(7.5, 1.0)
F (t)
6.96
J(1.6)
G (t)
6.21
J(1.4)
H (m)
3.65
I (t)
2.02
J(1.6)
C (td)
7.56
J(7.4, 1.2)
f1 (ppm)

B.

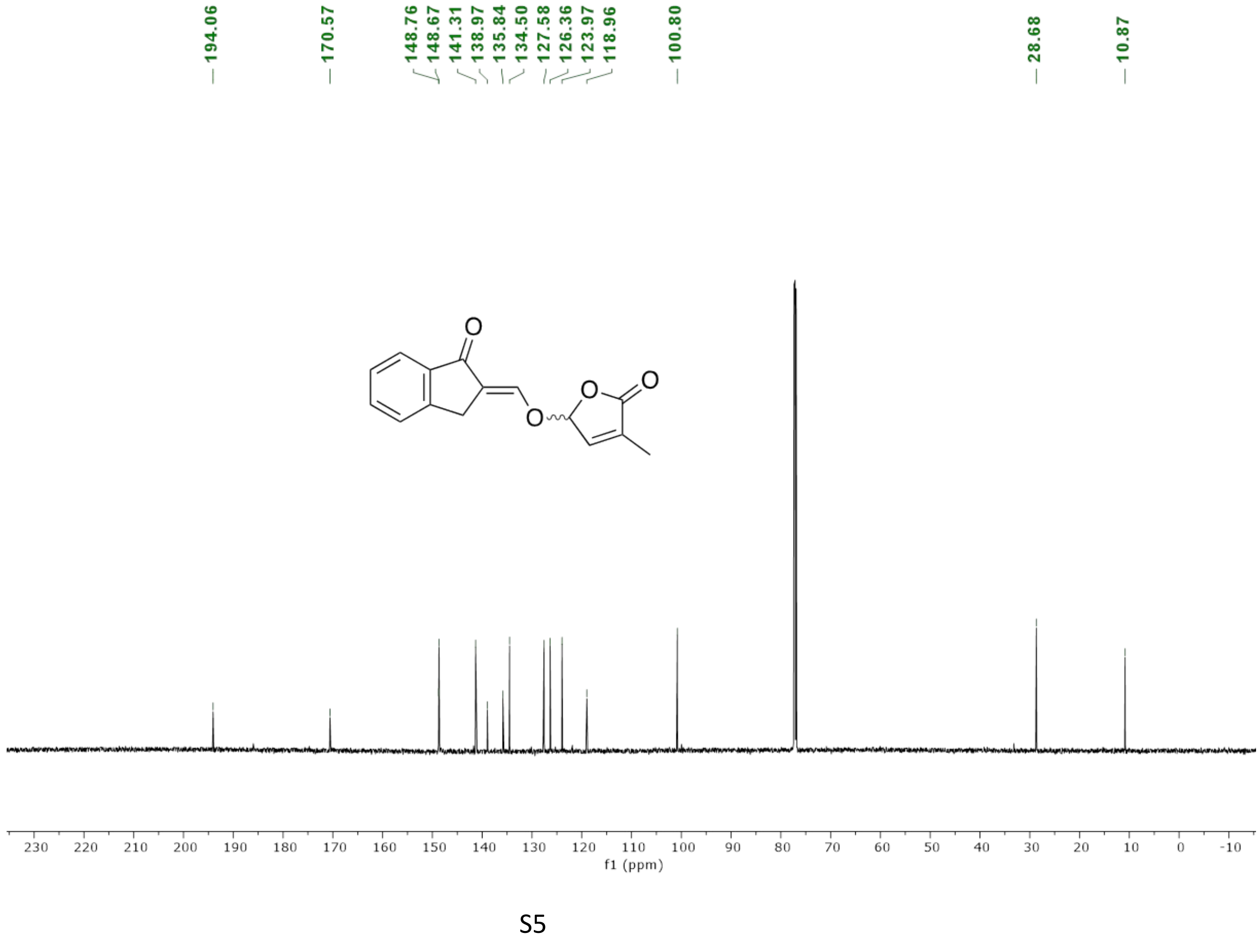
194.06
170.57
148.76
148.67
141.31
138.97
135.84
134.50
127.58
126.36
123.97
118.96
100.80
28.68
10.87
f1 (ppm)

Figure S1. Spectroscopic properties of IND including (*A.*) H1 and (*B.*) C13 NMR analysis. GR24, EGO10 and 4-Br-debranone were purchased from Strigolab srl. Purity of compounds has been assessed by the company by HPLC quality control report. IND was synthesized according to procedure reported in[14]. Spectroscopic properties were coherent with those reported in the paper.

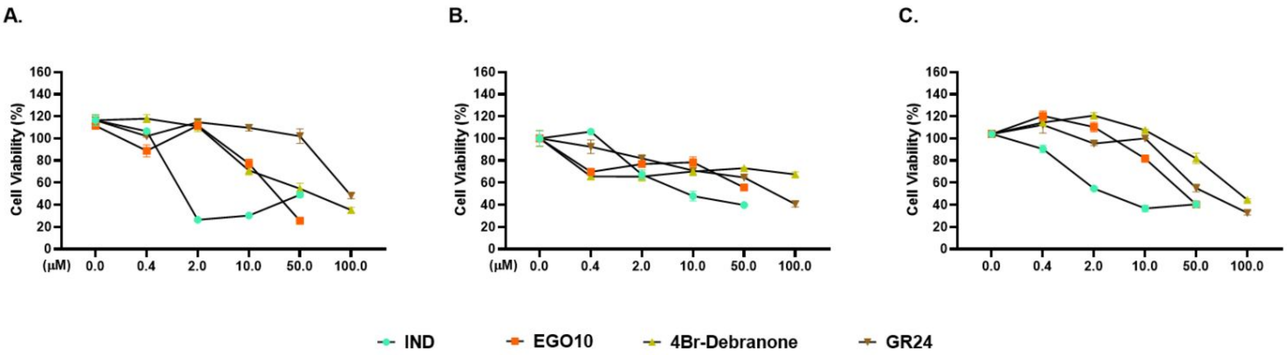

Figure S2. Effects of the SL analogs on the cell growth of *A.* A172 and *B.* U87 human glioblastoma and *C.* HUVEC endothelial cell lines at the dose range of 0.4-100 µM for 24 h. Data were represented as the mean ± SEM obtained from three independent experiments. SEM, Standard Error of Mean. C: Control (only DMSO).

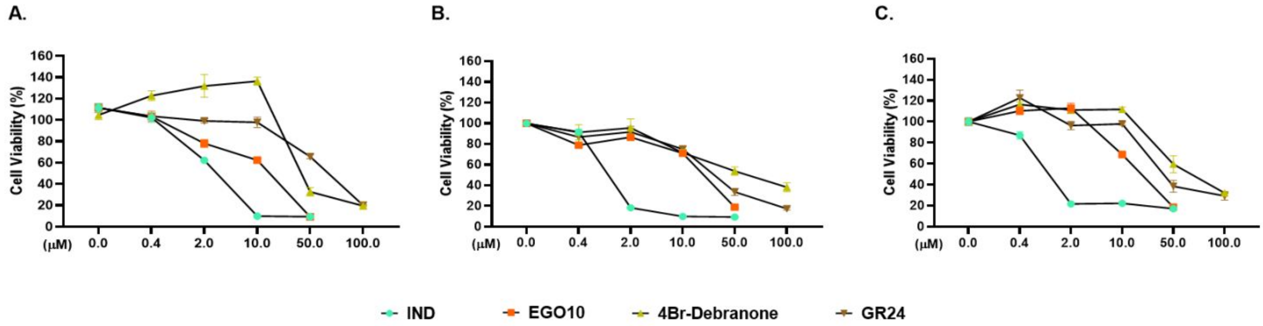

Figure S3. Effects of the SL analogs on the cell growth of *A.* A172 and *B.* U87 human glioblastoma and *C.* HUVEC endothelial cell lines at the dose range of 0.4-100 µM for 48 h. Data were represented as the mean ± SEM obtained from three independent experiments. SEM, Standard Error of Mean. C: Control (only DMSO).

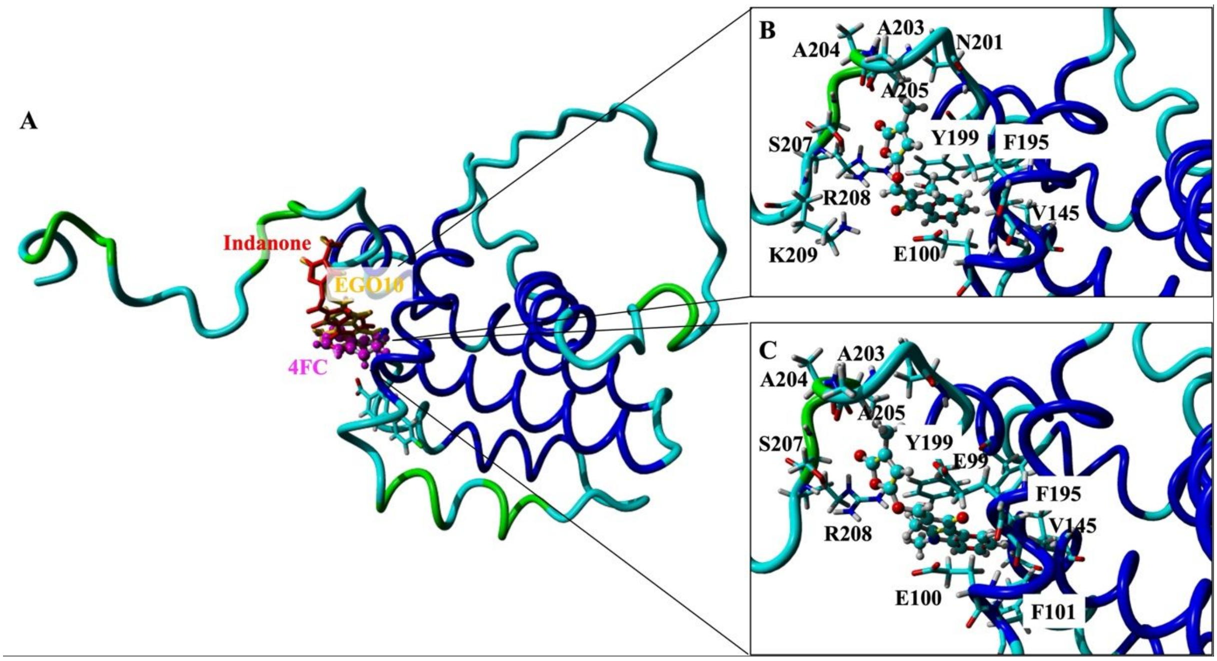

Figure S4. A) Ligands (indanone-derived SL (IND) in red, EGO10 in orange) positioned in the same binding pocket after molecular docking simulations were visualized, superimposed with the crystal structure ligand, 4FC (magenta). Zoomed in 3-D view to the binding interactions for IND (B) and EGO10 (C) molecules.

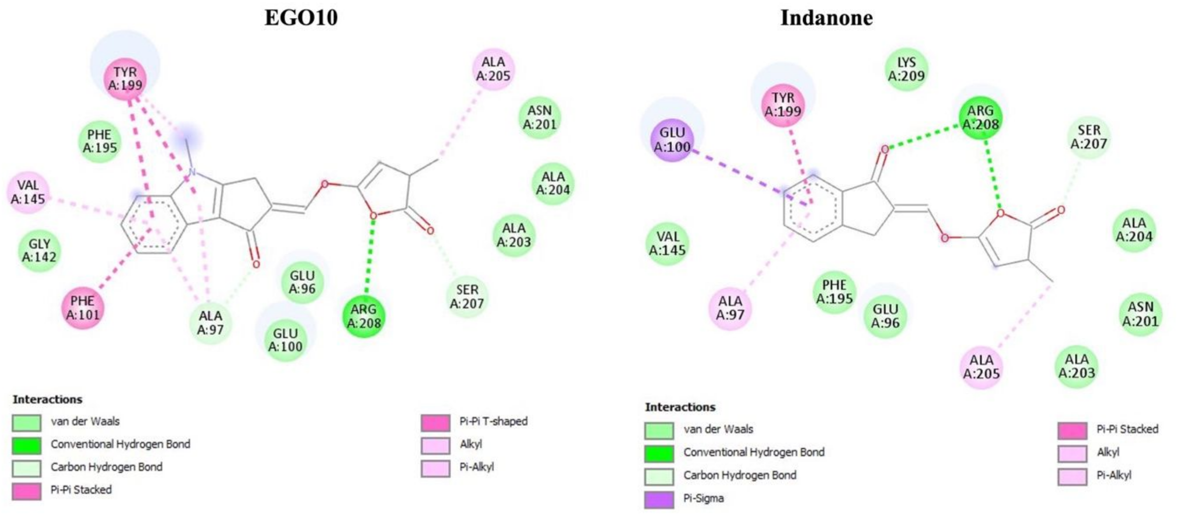

Figure S5. 2-D interaction schemes for EGO10 and IND with Bcl-2 protein obtained from molecular docking simulations.

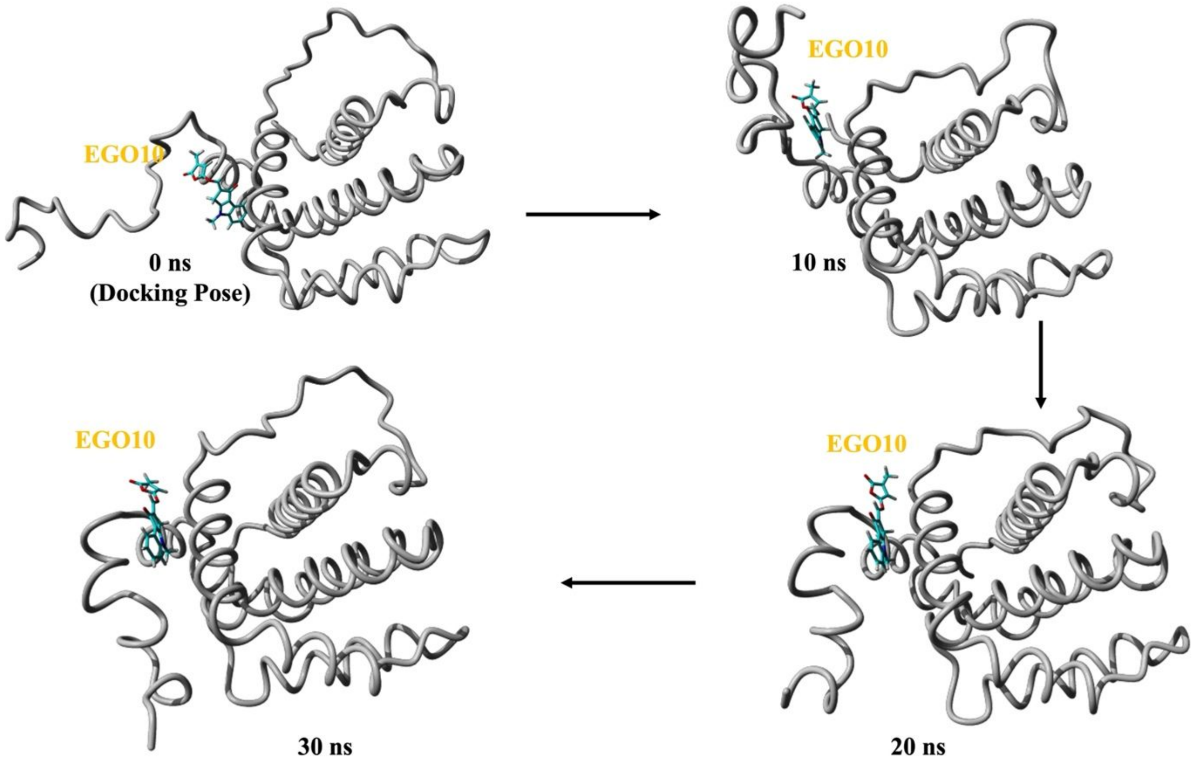

Figure S6. Time evolution of EGO10-Bcl2 complexes throughout MD simulations. EGO10 stayed at the very similar binding site obtained from molecular docking simulations throughout MD simulations.

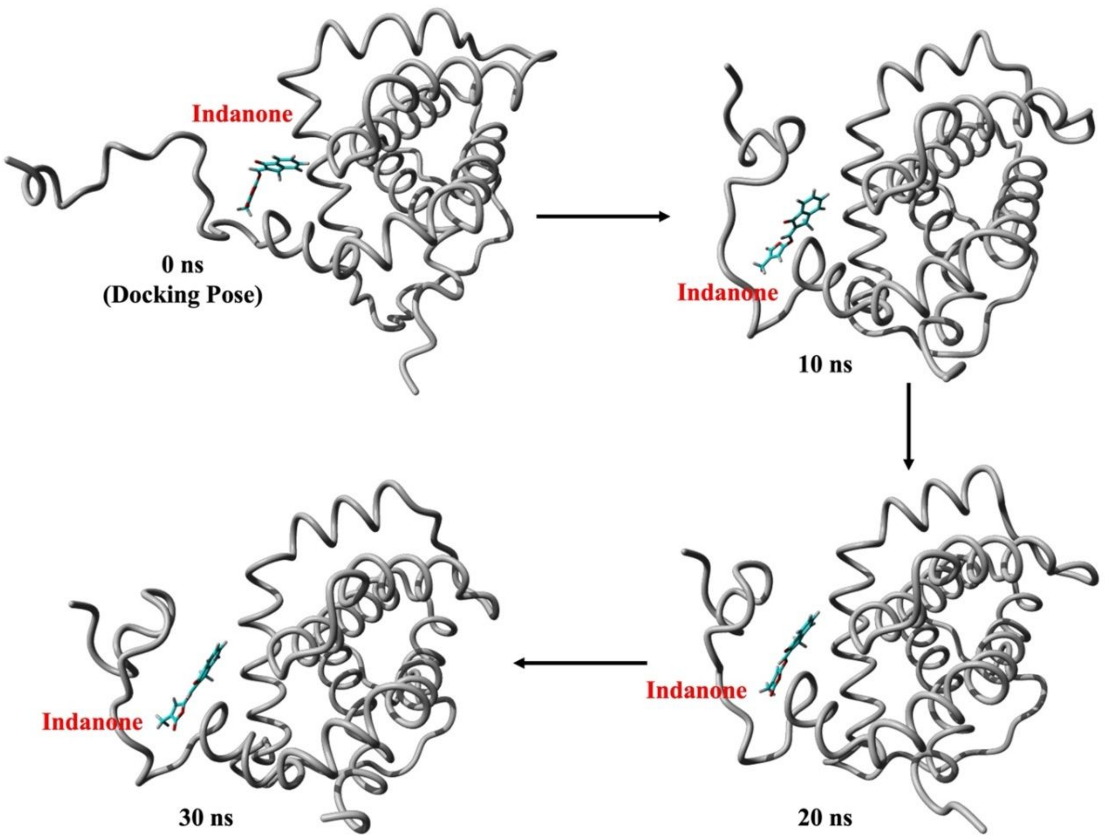

Figure S7. Time evolution of IND-Bcl2 complexes throughout MD simulations. IND stayed at the very similar binding site obtained from molecular docking simulations throughout MD simulations.

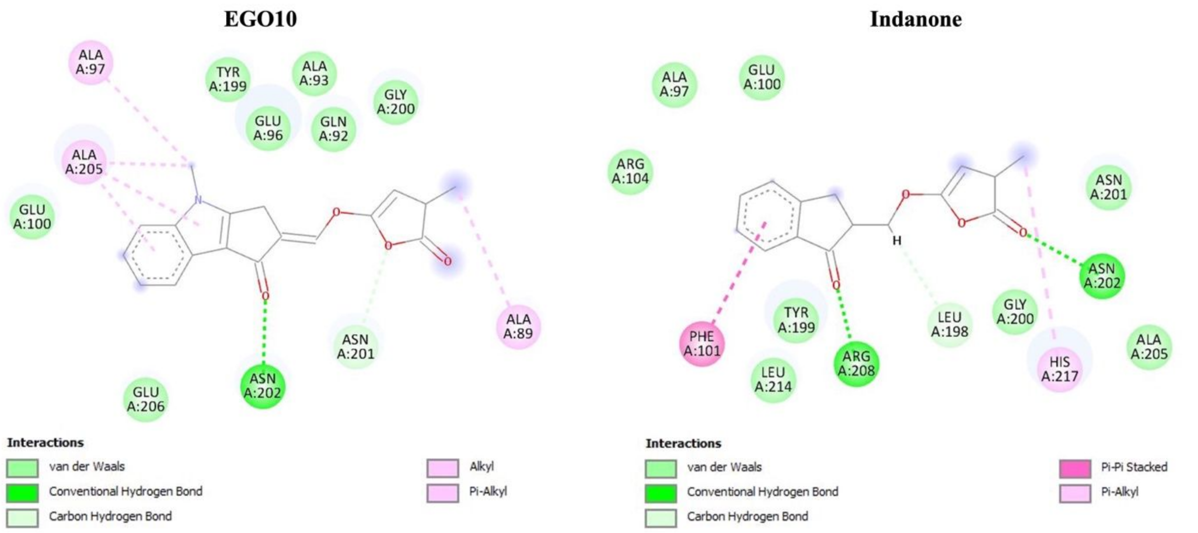

Figure S8. 2-D interaction schemes for EGO10 and IND with Bcl-2 protein obtained from molecular dynamics simulations.
